# Myelitis in congenital toxoplasmosis: An unusual presentation

**DOI:** 10.7705/biomedica.7829

**Published:** 2026-10-16

**Authors:** Martha Africano, Yuly Bayona, Diego Bravo, Silvia Suárez-Mantilla

**Affiliations:** 1 Departamento de Pediatría, Clínica Materno Infantil San Luis, Bucaramanga, Colombia Departamento de Pediatría Clínica Materno Infantil San Luis Bucaramanga Colombia; 2 Grupo de Investigación PAIDÓS, Departamento de Pediatría, Facultad de Salud, Universidad Industrial de Santander, Bucaramanga, Colombia Universidad Industrial de Santander Departamento de Pediatría Facultad de Salud Universidad Industrial de Santander Bucaramanga Colombia

**Keywords:** Myelitis, toxoplasmosis, congenital, toxoplasma, infant, premature, neonatal diseases, infant, newborn, diseases/diagnosis, toxoplasmosis, cerebral, Colombia, mielitis, toxoplasmosis congénita, toxoplasma, recién nacido prematuro, enfermedades del recién nacido/diagnóstico, toxoplasmosis cerebral, Colombia

## Abstract

A late preterm newborn presented with paresis of the lower limbs with right-sided predominance, ipsilateral patellar areflexia and left-sided hyporeflexia, suggestive of myelitis, which was confirmed by magnetic resonance imaging. The diagnosis of congenital toxoplasmosis was confirmed through the presence of specific immunoglobulins for *Toxoplasma gondii* in both the mother and the infant. The patient was treated with pyrimethamine, sulfadiazine, and systemic corticosteroids, but showed no clinical improvement.

## Resumen

Se trata de un recién nacido prematuro tardío que presentó paresia de las extremidades inferiores con predominio del lado derecho, arreflexia rotuliana ipsilateral e hiporreflexia en el lado izquierdo, hallazgos sugestivos de mielitis, confirmada mediante resonancia magnética de columna. El diagnóstico de toxoplasmosis congénita se estableció mediante la detección de inmunoglobulinas específicas para *Toxoplasma gondii* tanto en la madre como en el lactante. El paciente recibió tratamiento con pirimetamina, sulfadiacina y corticosteroides sistémicos; sin embargo, no presentó una mejoría clínica significativa.

Congenital toxoplasmosis is an infectious disease caused by the transplacental transmission of the parasite *Toxoplasma gondii*, an intracellular parasite that affects up to one-third of the global population ^1^^,^^2^. It is considered a global public health issue, particularly in developing countries and tropical regions such as Colombia. In these areas, 50 to 60% of pregnant women have anti-*Toxoplasma* antibodies, and 0.6 to 3% of expectant mothers acquire the infection during pregnancy ^3^

Its clinical spectrum is broad, ranging from asymptomatic cases to severe manifestations, including hydrocephalus, brain calcifications, chorioretinitis, hepatosplenomegaly, cytopenia, and skin lesions ^4^. Symptoms are known to be more severe when the infection occurs during the first trimester of pregnancy ^5^.

This report presents the case of a patient with congenital toxoplasmosis and multisystemic involvement, including severe brain abnormalities (microcephaly, ventriculomegaly and cerebral calcifications) and spinal cord damage (extensive myelitis).

## Case report

A late preterm newborn, product of a second pregnancy, was born to a previously healthy mother who had inadequate prenatal care. The pregnancy was complicated by gestational diabetes managed with diet. The mother lived in an urban area from Aguachica (Cesar) and was a housewife. She reported having three pet cats, all older than one year; however, litter box hygiene was not strictly maintained. According to her socioeconomic conditions, she did not have access to potable water and reported consistently drinking boiled water. She also described maintaining a balanced diet, including the consumption of well-cooked meat. No other significant environmental risk factors were identified.

She remained asymptomatic throughout the entire pregnancy. At 8 weeks of gestation, laboratory tests showed negative *T. gondii* IgG (8.25 IU/ml); IgM testing was not performed for reasons unknown to the patient. ELISA testing for HIV-1 and HIV-2 was negative. Hepatitis B surface antigen (HBsAg) was negative, and the non-treponemal test for syphilis was also negative. The patient reported irregular attendance to prenatal check-up due to personal difficulties in keeping appointments, as well as delays in receiving timely medical care.

At 33 weeks of gestational age, the mother consulted due to the absence of fetal movements. No further prenatal studies had been conducted. A transabdominal ultrasound was performed, showing biometry for 33.3 weeks, an amniotic fluid index of 19 cc, increased periventricular echogenicity associated with periventricular microcalcifications and ventriculomegaly. Additional maternal laboratory tests revealed positive IgG and IgM antibodies for *T. gondii*, while the rest of the prenatal infectious screening was negative.

Serological testing was performed using electrochemiluminescence immunoassays from Roche Diagnostics™. According to the manufacturer’s instructions, IgM was considered positive with a cut-off index > 1 and IgG ≥ 30 IU/ml. At that moment, treatment was started with spiramycin, according to the Colombian guideline for gestational toxoplasmosis ^3^.

The baby was born via spontaneous vaginal delivery at 36 weeks and 5 days of gestation without complications. He developed respiratory distress and required management with non-invasive mechanical ventilation. On physical examination, microcephaly and hepatosplenomegaly were noted. Additionally, there was paresis of the lower limbs with right-sided predominance, ipsilateral patellar areflexia, and left-sided hyporeflexia, leading to suspicion of associated myelitis.

Further blood tests revealed positive toxoplasma IgM (cut-off index 68.4), positive toxoplasma IgG (650 IU/ml), moderate thrombocytopenia and elevated liver enzymes. The cerebrospinal fluid showed elevated protein level and low glucose. Brain computed tomography revealed multiple abnormalities, including bilateral periventricular and cortico-subcortical calcifications, parenchymal volume loss, and ventriculomegaly (figure 1).

**Figure 1.**
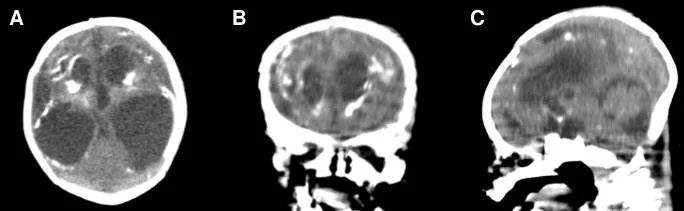
Computed tomography. **A.** Axial; **B.** Coronal; **C.** Sagittal. Non-contrast computed tomography images show extensive periventricular and cortico-subcortical calcifications, moderate ventriculomegaly and brain volume loss.

Ophthalmology was consulted, and panuveitis was reported. The retina could not be assessed anatomically due to vitreous opacity. Magnetic resonance imaging of the spine, with and without contrast, was performed, confirming myelitis (figure 2).

**Figure 2.**
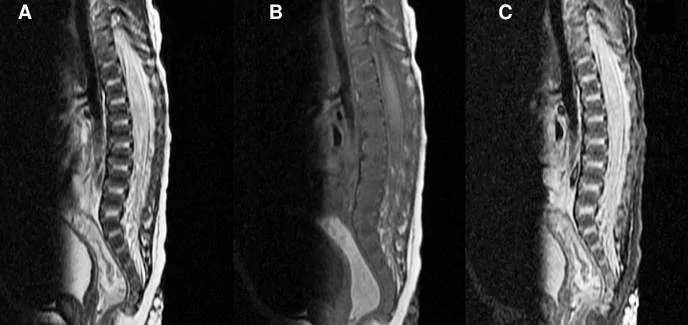
Spinal magnetic resonance imaging, sagittal; **A.** T2-weighted, **B.** T1-weighted, and **C.** STIR (short tau inversion recovery) images show an expansile hyperintense lesion with some isointense areas from T10 to L1, suggestive of myelitis.

Treatment was initiated with pyrimethamine and sulfadiazine, along with folinic acid and systemic corticosteroids. While undergoing treatment, the patient experienced clinical seizures, which were controlled with phenobarbital. Additionally, urinary retention was detected, leading to the initiation of intermittent catheterization. Unfortunately, the patient’s condition remained stationary, with no improvement in lower limb mobility.

## Ethical aspects

The case has the patient’s parents informed consent in accordance with the formats established by British Medical Journal (BMJ) Case Reports. In addition to complying with Colombian resolution 8430 of October 4, 1993, which guarantees the protection of the subjects' data privacy rights. The study was approved by the *Clínica Materno-Infantil San Luis* Research Ethics Committee (approval 04062019).

## Discussion

Congenital toxoplasmosis has an incidence of 0.2 cases per 1000 live births in the United States ^6^^,^^7^. In Colombia, the incidence of congenital toxoplasmosis, according to the World Heal Organization (WHO), is 1.8 cases per 1000 live births ^8^. A multicenter Colombian study on congenital toxoplasmosis incidence, including Bucaramanga, reported national cord blood positivity rates of 0.39% for IgM and 0.5% for IgA. Bucaramanga specifically showed 0.23% for IgM and 0.78% for IgA ^9^.

Most newborns with congenital toxoplasmosis are asymptomatic (80%), and present with mild and nonspecific symptoms such as fever, chills, myalgia, pharyngitis, lymphadenopathy, hepatosplenomegaly, and/or a diffuse nonpruritic maculopapular rash. Nevertheless, in one-third of cases, a severe clinical presentation is observed, including cerebral and ocular involvement ^2^.

The most frequent manifestations when the central nervous system is affected are intracranial calcifications and hydrocephalus, and ocular involvement commonly presents as chorioretinitis. Spinal cord involvement has been rarely reported ^10^^,^^11^.

Hydrocephalus symptoms may be the first clinical sign of brain involvement. Usually, it is secondary to obstruction of the Sylvian aqueduct. Clinically, it may present with seizure episodes, muscle fasciculations, and opisthotonos ^12^.

Infectious myelitis is a rare condition, with an incidence of 1.3 to 8 cases per 1 000 000 inhabitants. However, parasites such as *T. gondii* can cause it ^12^. Spinal cord or bulbar involvement may be revealed through limb paralysis, feeding difficulties, and respiratory distress. ^12^. This condition typically occurs in immunocompromised patients and in co-infections such as human immunodeficiency virus, tuberculosis, and leukemia. Fewer than five cases of congenital toxoplasmosis with spinal cord involvement have been reported in the literature, and according to our research, this is the first one published in Colombia.

In summary, spinal cord involvement should be considered in patients with congenital toxoplasmosis who present with paraparesis and sphincter dysfunction. Severe cases like this highlight the importance of ensuring high-quality prenatal care to enable the prevention and early detection of such diseases, thereby reducing their impact.

This case highlights the consequences of non-adherence to clinical management guidelines, consistent with findings from several studies conducted in Colombia ^13^^-^^15^. It also underscores the importance of primary and secondary prevention of gestational toxoplasmosis.

Since 2013, a national guideline has been in place that mandates systematic screening from the first prenatal visit. This screening, now fully guaranteed by the national health system without barriers to access, includes testing for *T. gondii* IgG and IgM antibodies, followed by monthly follow-up according to the results and a clearly established management algorithm ^14^^,^^15^. This approach represents the most cost-effective strategy to improve the timely diagnosis and treatment of both gestational and congenital toxoplasmosis, thereby reducing associated morbidity and mortality ^14^^,^^15^.

This impact has been demonstrated in the study by Mejía-Oquendo *et al*., which evaluated outcomes before and after the implementation of the Colombian clinical practice guidelines for congenital toxoplasmosis in 2013. Prior to their implementation, 27.3% of cases lacked a prenatal diagnosis; however, after the introduction of the guidelines, all mothers were diagnosed during pregnancy ^16^.

Addressing barriers within the healthcare system is also essential, including limited access to diagnostic testing, insufficient training and continuing education for healthcare personnel, and the need to strengthen the physician-patient relationship to improve adherence to recommended procedures and follow-up ^13^^-^^16^.
